# Systematic analysis of genetic variants in patients with essential tremor

**DOI:** 10.1002/brb3.1100

**Published:** 2018-09-05

**Authors:** Lamei Yuan, Xiong Deng, Zhi Song, Sheng Deng, Wen Zheng, Ping Mao, Hao Deng

**Affiliations:** ^1^ Center for Experimental Medicine The Third Xiangya Hospital, Central South University Changsha China; ^2^ Department of Neurology The Third Xiangya Hospital, Central South University Changsha China; ^3^ Department of Pharmacy Xiangya Hospital, Central South University Changsha China; ^4^ Department of Nursing Education The Third Xiangya Hospital, Central South University Changsha China

**Keywords:** association, essential tremor, genetics, the *MC1R* gene, variant

## Abstract

**Background:**

Essential tremor (ET), a prevalent neurological disorder, is featured by postural and kinetic tremors in upper limbs. Studies of twins and families indicate an important role for genetic factors in ET development. There are substantial overlaps between ET and Parkinson's disease (PD). The aim of this study was to examine the possible roles of genetic variants in ET development.

**Methods:**

A total of 200 Han Chinese ET patients and 432 ethnically matched normal controls were enrolled, and genetic analysis of 23 variants in 15 genes was performed.

**Results:**

Genotypic and allelic frequencies of the melanocortin 1 receptor gene (*MC1R*) variant rs34090186 showed statistically significant differences in ET patients and controls (*p* = 0.027 and 0.028, odds ratio = 2.789 and 2.744, 95% confidence interval: 1.084–7.179 and 1.075–7.005). No statistically significant difference was revealed in either genotypic or allelic distributions of other variants or haplotypes (all *p* > 0.05).

**Conclusions:**

The discrepancies found in this study indicate the variant rs34090186 in the *MC1R* gene, some variants of which were reported to be related to increased risk of PD and melanoma, may play a risk role in ET, confirming a potential association between ET and PD. Evidence supporting ET‐PD link will continue to accumulate and improve our understanding of any underlying mechanisms for both disorders.

## 1 INTRODUCTION

Essential tremor (ET) and Parkinson's disease (PD), two adult common tremor disorders, are among the most frequent neurological disorders having a crude prevalence of 0.4% and 0.3%, respectively. Prevalence increases with age (de Lau & Breteler, 2006; Louis & Ferreira, 2010; Thenganatt & Jankovic, 2016). Typical features of ET are postural and kinetic tremors during voluntary motion (action tremors), worsening with movement, while typical PD tremors primarily occur at rest (rest tremors) (Thenganatt & Jankovic, 2016; Yuan, Song, Deng, Zheng, Yang, et al., 2016). Both types of tremors can present in two disorders and be accompanied by other overlapping features (Thenganatt & Jankovic, 2016). ET patients have a four‐ to fivefold increased risk of PD development compared to controls (Unal Gulsuner et al., 2014). Although the association is as yet controversial, the coexistence of those two conditions is more than coincidental. Epidemiologic, clinical, imaging, genetic, and pathologic features support a conclusion of a relationship between ET and PD, showing possible common mechanisms shared in two tremor disorders (Jimenez‐Jimenez et al., 2013; Louis, Clark, & Ottman, 2016; Thenganatt & Jankovic, 2016). In this scenario, association studies performed in PD patients are extended to studies in ET cases (He et al., 2016; Shalaby & Louis, 2016; Unal Gulsuner et al., 2014; Yuan, Song, Deng, Zheng, Guo, et al., 2016; Yuan, Song, Deng, Zheng, Yang, et al., 2016).

At least ten loci, including chromosome 3q13.31 (*ETM1*), 2p24.1 (*ETM2*), 6p23 (*ETM3*), 16p11.2 (*ETM4*), 11q14.1 (*ETM5*), 2p13.1, 1p13.3, 17q23.3, 5q35, and 3p22.2, have been reported to be related to ET development. By linkage analysis, whole‐exome sequencing and genomewide association studies, several genes have been identified to be associated with monogenic inherited ET or ET susceptibility, such as the FUS RNA‐binding protein gene (*FUS*), the HtrA serine peptidase 2 gene (*HTRA2*), the sortilin 1 gene (*SORT1*), the teneurin transmembrane protein 4 gene (*TENM4*), the sodium voltage‐gated channel alpha subunit 4 gene (*SCN4A*), *SCN11A*, the dopamine receptor D3 gene (*DRD3*), the leucine‐rich repeat and Ig domain containing 1 gene (*LINGO1*), the solute carrier family 1 member 2 gene (*SLC1A2*), the heme oxygenase 1 gene (*HMOX1*), and *HMOX2*. However, further confirmation is needed (Hicks et al., 2016; Jimenez‐Jimenez et al., 2013; Kuhlenbaumer, Hopfner, & Deuschl, 2014; Leng, Qi, Zhou, & Wang, 2017; Tio & Tan, 2016; Yuan, Song, Deng, Zheng, Yang, et al., 2016). Variants of some genes, including the synuclein alpha gene (*SNCA*), *LINGO1*,* LINGO2*,* HTRA2*, the leucine‐rich repeat kinase 2 gene (*LRRK2*), and the DnaJ heat‐shock protein family (Hsp40) member C13 gene (*DNAJC13*), have been shown to exert either a risk or protective effect on ET and PD, or to be responsible for both disorders (Chao et al., 2015; Tio & Tan, 2016; Unal Gulsuner et al., 2014). Genetic or environmental modifying factors alone, or combination of these two factors as possible confounding variables, may contribute to the ET etiopathogenesis (Jimenez‐Jimenez et al., 2013; Louis, 2018; Tio & Tan, 2016).

This study evaluated 23 variants of 15 genes in 200 patients with ET and 432 ethnically matched normal controls, in order to explore any associations between the variants and developing ET, and to reveal any potential genetic links between ET and PD.

## 2 MATERIALS AND METHODS

### 2.1 Participants

Two hundred unrelated Han Chinese patients with ET (male/female: 100/100) from mainland China participated in the genetic study. There were 432 unrelated normal controls of similar gender, age, race, and residence (male/female: 216/216, age 52.3 ± 15.6 years). Of the 200 patients, the age, in years, at sample collection was 50.8 ± 15.3, and at ET onset was 41.5 ± 18.6. ET patients were consecutively enrolled from the Department of Neurology, the Third Xiangya Hospital of Central South University, Changsha, China. Two senior independent neurologists using the internationally accepted clinical criteria made the clinical diagnoses (Deuschl, Bain, & Brin, 1998; Louis, Ford, Lee, Andrews, & Cameron, 1998). Confusing conditions, such as PD, dystonia, and psychogenic tremor, or any positive family history of these conditions, were excluded from both the patient group and the control group (Yuan, Song, Deng, Zheng, Yang, et al., 2016). Some patients had been previously studied to explore potential associations between gene variants and ET risk. Half of the patients were evaluated for coding mutations in the *LINGO4* gene and *LINGO1* gene. Seventy‐four percent (148/200) of the cases were screened for the *FUS* gene coding variants, and all patients were tested for seven variants, including rs1721100, rs1989754, rs10089600, rs12720208, rs17550360, rs3758549, and rs4919621 (Chen, Song, et al., 2017; Yuan, Song, Deng, Zheng, Yang, et al., 2016; Zheng et al., 2013). The study was conducted in adherence to the Declaration of Helsinki guidelines and approved by the Institutional Review Board of the Third Xiangya Hospital, Central South University. Written informed consent was obtained from all subjects before having peripheral venous blood sampled for genomic DNA extraction.

### 2.2 Variants

A genetic overlap between PD and ET was posited, and variants in genes assumed to be related to PD were investigated in this study. Variants of reported PD‐ and ET‐associated genes in the published literature or paralogs were selected based on the criteria: the recorded minor allele frequencies of no less than 5% in the Single Nucleotide Polymorphism database (dbSNP) and predicted deleterious effects by bioinformatics (Kumar, Henikoff, & Ng, 2009; Schwarz, Cooper, Schuelke, & Seelow, 2014; Yuan, Song, Deng, Zheng, Guo, et al., 2016).

### 2.3 Variant genotyping

The previously described standard phenol–chloroform extraction method was applied for the isolation of genomic DNA from the peripheral blood cells (Deng et al., 2016). Matrix‐assisted laser desorption/ionization time‐of‐flight mass spectrometry was used to genotyping, which was performed by Bioyong Technologies (Beijing, China) according to manufacturers’ instructions (Buetow et al., 2001; Johansen, Andersen, Borsting, & Morling, 2013). Sequenom Assay Design 3.1 software was used to design locus‐specific PCR amplification and single‐base extension primers. A Sequenom MassARRAY system was applied to test the primer quality. Before the mass spectrometry for genotyping, multiplex locus‐specific PCR amplification, purification of PCR products, and single‐base extension were carried out as previously described (He et al., 2016; Yuan, Song, Deng, Zheng, Yang, et al., 2016). Genotype of each variant was determined using a MassARRAY Typer 4.0 software (Sequenom) by analyzing the mass spectrometric results. Sample status of patient or control was inaccessible to the investigators. Twenty‐microliter PCR amplifications were performed with 40 ng of genomic DNA and 10 μmol of each primer (forward and reverse primers) using a 2× power Taq PCR MasterMix (BioTeke Co., Beijing, China) in 8% of randomly selected samples. Sanger sequencing of PCR products was conducted using an 8‐capillary 3500 genetic analyzer (Applied Biosystems Inc., Foster City, CA, USA) to test the reliability and the accuracy of mass spectrometric genotyping (Deng, Le, & Jankovic, 2004; Yuan, Song, Deng, Zheng, Guo, et al., 2016; Zheng et al., 2013).

### 2.4 Statistical analysis

Hardy–Weinberg equilibrium was used to estimate the normal deviation of genotypes in control cohort (He et al., 2016; Yuan, Song, Deng, Zheng, Guo, et al., 2016). Pearson's *χ* ^2^ test for statistical differences in genotypic and allelic distributions and haplotype analysis in two groups were evaluated in the Predictive Analytics Software Statistics 18 software (SPSS, Chicago, IL, USA) and Online SHEsis program (https://analysis.bio-x.cn). *p*‐Values, odds ratios, and 95% confidence intervals were generated for statistical results. The discrepancies are statistically significant with the obtained two‐sided *p*‐values less than 0.05 (Li et al., 2009; Yuan, Song, Deng, Zheng, Guo, et al., 2016).

## 3 RESULTS

All the evaluated 23 variants in this study were examined in the 200 Han Chinese ET patients and the 432 healthy controls with specific primers (Table S1). The Hardy–Weinberg equilibrium for each variant was revealed in controls (all *p* > 0.05). Eighteen variants showed monomorphism (no alternative genotype), including rs2227843, rs2227849, rs28924121, rs72470545, rs10115304, rs73672607, rs74942016, rs80127039, rs3207505, rs34184838, rs375681722, rs538881762, *TENM4* c.4100C>A (p.T1367N), rs75932628, rs74654177, rs13071187, rs2230149, and rs35693565. Genotypic and allelic distributions for the other five variants in ET patients and controls appear in Table 1. Statistically significant difference of genotypic distribution between ET patients and controls was only observed in the melanocortin 1 receptor gene (*MC1R*) variant rs34090186 (*χ* ^2^ = 4.897, *p* = 0.027, odds ratio = 2.789, 95% confidence interval: 1.084–7.179). The patient group had a higher A allele frequency (*χ* ^2^ = 4.826, *p* = 0.028, odds ratio = 2.744, 95% confidence interval: 1.075–7.005) compared to the control group. No statistically significant differences were found in genotypic or allelic frequencies of the other variants, including rs33932559, rs2254562, rs2076485, and rs7757931 (all *p* > 0.05, Table 1). No evidence of potential association with ET was found in haplotypes of the enrolled four variants, rs33932559–rs34090186 (*MC1R*) or rs2076485–rs7757931 (the ubiquitin D gene, *UBD*), as all *p*‐values were greater than 0.05 (Table 2).

**Table 1:** Genotypic and allelic distributions of gene variants in Han Chinese patients with essential tremor and ethnically matched controls

dbSNP ID	Gene	Genotype/Allele	Patients (freq)	Controls (freq)	*χ* ^2^ value	*p*‐Value	OR (95% CI)
rs33932559	*MC1R*	TT	181 (0.905)	399 (0.924)			
		TC	19 (0.095)	33 (0.076)			
		CC	0	0	0.627	0.428	1.269 (0.703–2.292)
		T	381 (0.953)	831 (0.962)			
		C	19 (0.047)	33 (0.038)	0.600	0.439	1.256 (0.705–2.237)
rs34090186	*MC1R*	GG	190 (0.950)	424 (0.981)			
		GA	10 (0.050)	8 (0.019)			
		AA	0	0	4.897	**0.027**	2.789 (1.084–7.179)
		G	390 (0.975)	856 (0.991)			
		A	10 (0.025)	8 (0.009)	4.826	**0.028**	2.744 (1.075–7.005)
rs2254562	*SYNJ1*	TT	67 (0.335)	170 (0.394)			
		TC	102 (0.510)	201 (0.465)			
		CC	31 (0.155)	61 (0.141)	1.997	0.368	
		T	236 (0.590)	541 (0.626)			
		C	164 (0.410)	323 (0.374)	1.509	0.219	1.164 (0.913–1.483)
rs2076485	*UBD*	TT	132 (0.660)	266 (0.615)			
		TC	53 (0.265)	148 (0.343)			
		CC	15 (0.075)	18 (0.042)	5.922	0.052	
		T	317 (0.792)	680 (0.787)			
		C	83 (0.208)	184 (0.213)	0.049	0.825	1.033 (0.772–1.383)
rs7757931	*UBD*	CC	174 (0.870)	358 (0.828)			
		CA	25 (0.125)	69 (0.160)			
		AA	1 (0.005)	5 (0.012)	2.007	0.367	
		C	373 (0.933)	785 (0.909)			
		A	27 (0.067)	79 (0.091)	2.039	0.153	0.719 (0.457–1.133)

Notes

Statistically significant *p‐*values are shown in bold.

CI: confidence interval; dbSNP: Single Nucleotide Polymorphism database; *MC1R*: the melanocortin 1 receptor gene; OR: odds ratio; *SYNJ1*: the synaptojanin 1 gene; *UBD*: the ubiquitin D gene.

**Table 2:** Haplotype analysis in patients with essential tremor and controls

dbSNP ID	Gene	Haplotype	Patient (%)	Control (%)	*χ* ^2^ value	*p‐*Value	OR (95% CI)
rs33932559–rs34090186	*MC1R*	T‐G	92.9	95.3	0.641	0.423	0.788 (0.439–1.414)
		C‐G	4.6	3.7	0.641	0.423	1.269 (0.707–2.277)
		T‐A	2.4	0.8	—	—	—
		C‐A	0.1	0.1	—	—	—
rs2076485–rs7757931	*UBD*	T‐C	72.5	70.4	0.320	0.571	1.079 (0.829–1.406)
		C‐C	20.7	20.5	0.002	0.968	1.006 (0.751–1.348)
		T‐A	6.7	8.3	1.021	0.312	0.790 (0.499–1.250)
		C‐A	0.0	0.8	—	—	—

Notes

All those haplotypes with frequency less than 0.03 are not considered in analysis.

CI: confidence interval; dbSNP: Single Nucleotide Polymorphism database; *MC1R*: the melanocortin 1 receptor gene; OR: odds ratio; *UBD*: the ubiquitin D gene.

## 4 DISCUSSION

Compared to controls, Asian PD patients were reported to be five to 10 times more likely to develop ET (Tan, Lee, S, & Lum, 2008). High concordance in monozygotic twins, a positive family history, and familial aggregation of ET and PD indicate the important role of genetic factors in developing the conditions (Louis et al., 2016; Wirdefeldt, Gatz, Reynolds, Prescott, & Pedersen, 2011). The putative association between the potential risk variants of PD and the development of ET was investigated in ET patients and ethnically matched normal controls for a common genetic link, due to the considerable epidemiological, clinical, imaging, genetic, and neuropathological overlaps between ET and PD (Thenganatt & Jankovic, 2016). PD patients are reported to have an increased odds of melanoma, and the *MC1R* gene variants in humans are related to an increased risk of melanoma and PD (Chen, Chen, et al., 2017; Shalaby & Louis, 2016). This study suggests that the *MC1R* rs34090186 variant may be associated with ET in a Han Chinese population.

The *MC1R* gene (OMIM 155555), located in chromosome 16q24.3, was initially known as an intronless gene spanning over 3 kb. It encodes a G‐protein‐coupled receptor with seven transmembrane domains, consisting of 317 amino acids (Garcia‐Borron, Abdel‐Malek, & Jimenez‐Cervantes, 2014). The encoded protein is primarily expressed in melanocytes and is also detected in nonmelanocytic cells including neurons, where it exerts a neuroprotective effect (Catania, 2008; Garcia‐Borron et al., 2014; Tell‐Marti et al., 2015). It involves melanogenesis regulation and induces the downstream expression of the nuclear receptor subfamily 4 group A member 2 gene (*NR4A2*), which is a transcription factor primarily expressed in the central nervous system, including dopaminergic neurons related to PD pathogenesis and is essential for dopaminergic phenotype induction and late dopaminergic precursor neuron survival (Garcia‐Borron et al., 2014; Saucedo‐Cardenas et al., 1998; Tell‐Marti et al., 2015). The *MC1R* gene is highly polymorphic in humans, and some identified variants are relevant to pigmentary phenotype, melanoma, oculocutaneous albinism, and PD (Garcia‐Borron et al., 2014; Tell‐Marti et al., 2015).

Generally, individuals with PD have an overall lower incidence of most types of cancers. However, melanoma, known as a malignant tumor of melanin‐producing cells in skin, is more frequent than expected in the presence of PD. Patients with melanoma are more likely to have PD (Chen, Chen, et al., 2017; Devine, Plun‐Favreau, & Wood, 2011). Individuals with a positive family history of PD or melanoma are likely to confer a higher risk of development of the other, suggesting a common genetic susceptibility (Disse, Reich, Lee, & Schram, 2016). The *MC1R* gene has been reported to mediate a potential common pathogenic mechanism for both conditions (Chen, Chen, et al., 2017). This association between neurological disorders and melanoma may not be confined to PD, but may extend to other disorders such as ET, which is known to share a series of features and genetic risk factors with PD (Shalaby & Louis, 2016; Thenganatt & Jankovic, 2016).

Using a case–control methodology in a well‐defined Han Chinese cohort, we demonstrated a potential association of a single nucleotide variant in a known risk gene of PD, *MC1R* rs34090186 (c.200G>A, p.R67Q), with ET. This indicates a common underlying mechanism or potential link between PD and ET, at least in our Han Chinese population. Several *MC1R* gene variants, not including rs34090186, were found to contribute to the PD development, suggesting a converging and distinguishable genetic basis for these two disorders (He et al., 2016; Shi et al., 2016; Tell‐Marti et al., 2015). Due to the limited sample size, high genetic and clinical heterogeneity, and the rarity of genotype or allele in the Han Chinese population, further confirmatory and additional studies are warranted to conclusively establish any definite relationships between the variant and these conditions and explore any underlying mechanisms, which might usher in a new era of targeted therapeutic strategies for disorders, including PD and ET.

## CONFLICT OF INTEREST

None declared.
