# Supplementary material for: Systematic analysis of genetic variants in patients with essential tremor
Source: Brain Behav. 2018 Sep 5;8(10):e01100. doi: 10.1002/brb3.1100 (PMC6192404; doi:10.1002/brb3.1100)
Supplement: Supplementary file 1 [file BRB3-8-e01100-s001.doc]

**TABLE S1 Primer sequences used for locus-specific PCR amplification and single-base extension**

| **dbSNP ID** | **Gene^a^** | **Prediction^b^** | **Forward primer sequence^c^ (5'→3')** | **Reverse primer sequence^c^ (5'→3')** | **Product size** | **Extending primer sequence^c^ (5'→3')** |
| --- | --- | --- | --- | --- | --- | --- |
| rs10115304 | *LMX1B* | T/B/DC | acgttggatgACAGCGATACCTCCTTAACC | acgttggatgAGGAACTCTGCATGGAGTAG | 117 bp | ggccGAGGAGCCGAGGAAGCAGTC |
| rs13071187 | *UBA7* | -/-/DC | acgttggatgAGTGGGCATGGTGAACTTCC | acgttggatgTAGCATTCTGGACTAGACCC | 113 bp | taTGGACACAGCCCTGCTC |
| rs2076485 | *UBD* | D/P/Po | acgttggatgTAAGACCAAGGTTCCTGTGC | acgttggatgGTAAGGTGGATGGTCTTCTC | 114 bp | gggtATGGTCTTCTCTTTGTCA |
| rs2227843 | *DRD5* | D/P/DC | acgttggatgATGCAGAGAACTGTGACTCC | acgttggatgTAGGTCACGATCATGATGGC | 97 bp | CTCTTCCTCGCTCATCA |
| rs2227849 | *DRD5* | D/Pos/DC | acgttggatgGTCTTCATCGTGTCTCTGGC | acgttggatgTGGAGCACATGATGTCGAAG | 142 bp | gtttGCCGGTTACTGGCCCTTTG |
| rs2230149 | *UBA7* | D/P/Po | acgttggatgCTTTGACTTGGGCCTTACAG | acgttggatgTGGAATCCCGTAGTTCTGAC | 95 bp | CGCTACCACAAAGTCCACA |
| rs2254562 | *SYNJ1* | D/B/Po | acgttggatgCACCTTCCTTAGATCCAAGC | acgttggatgATTGGGCTTCTTATTTGAC | 120 bp | CCAAGCAAATTTACTATTATTTGT |
| rs28924121 | *FBXO6* | T/Pos/Po | acgttggatgTCTGGACAACAGCTCGGTAG | acgttggatgTCACCAACAGCAGCATTGTC | 126 bp | GTTCCTGGTCATCTTGG |
| rs3207505 | *NUCKS1* | D/B/DC | acgttggatgAAAATGTGCGCCAACAACGG | acgttggatgATACTCTCCTGGAATGGTGC | 127 bp | cccgGGAATGGTGCCTCATCCTCC |
| rs33932559 | *MC1R* | D/Pos/DC | acgttggatgGTGCTGCAGCAGCTGGACA | acgttggatgAGAAGATGGAGATGTAGCGG | 109 bp | GCAGGTGATCACGTCA |
| rs34090186 | *MC1R* | D/B/DC | acgttggatgAGGTGTCCATCTCTGACGG | acgttggatgTGAAGCAGTACATGGGTGAG | 120 bp | GGGTGAGTGCAGGTTC |
| rs34184838 | *PLA2G6* | D/B/DC | acgttggatgGCTCATAGATGTAGACCTCG | acgttggatgCTGGCCCGAACAGATTGAAC | 111 bp | agCATGCTGGATGAGGTCA |
| rs35693565 | *VPS41* | T/P/DC | acgttggatgACCCCTCAAGGCTACATATC | acgttggatgCCACATTTCGTGAGATCCAG | 95 bp | CCTTCTTCCCTCCGG |
| rs375681722 | *TENM4* | D/P/DC | acgttggatgTTGCTTAGCAGGAAGTGGTC | acgttggatgTGTCCTCGACAACAATGTGG | 114 bp | AGGTGCGCATTGTC |
| rs538881762 | *TENM4* | D/P/DC | acgttggatgTCTGGGCAGGATTCATATTC | acgttggatgCCTTGCCTACAGGTCTAAAG | 109 bp | AGGATTCATATTCATAACCCA |
| rs72470545 | *HTRA2* | D/P/DC | acgttggatgTCAGCATGGTGTACTCATCC | acgttggatgCCTAGTTCAAGGACACATGC | 114 bp | ACTCATCCATAAAGTCATCCTG |
| rs73672607 | *NRG1* | D/P/Po | acgttggatgCTTTCCTGGGCATACAGAAC | acgttggatgGGATTTCTTCCTGTGTCGAG | 114 bp | ccctGCAGCCAGTCTTGAGGCAACAC |
| rs74654177 | *UBA3* | D/Pos/DC | acgttggatgCGCTCGAGGAACTTCTTTAC | acgttggatgTTGCAGCTGAATTTACTGTG | 108 bp | CCCCACACCCACCATCA |
| rs74942016 | *NRG1* | D/Pos/Po | acgttggatgCATAACCGGCATCTGCATCG | acgttggatgAAGCTGTCTCTGCATGAAGG | 120 bp | gccgTGTGGTCGGCATCATGTGT |
| rs75932628 | *TREM2* | T/P/DC | acgttggatgCACAAGTTGTGCGTGCTGAC | acgttggatgCCTATGACTCCATGAAGCAC | 100 bp | GCACCAGGCCTTG |
| rs7757931 | *UBD* | D/B/Po | acgttggatgTAAGCTTTTTGACCCCTGCC | acgttggatgATGGCAGATTACGGCATCAG | 107 bp | TGGTCACCCTCCAATA |
| rs80127039 | *NRG1* | D/P/DC | acgttggatgTGAAACGACCCAAGAGTACG | acgttggatgTGCTGTCCACTTCCAATCTG | 119 bp | gttgGTTAAGAAACTCGCCAATAGC |
| *TENM4*  p.T1367N | *TENM4* | T/P/DC | acgttggatgTACAGTGGACAAGTTTGGGC | acgttggatgTGAGATCATTAGAGCCGAGC | 104 bp | tATCGATGCGTCTGATCATG |

B, benign; D, damaging; DC, disease causing; dbSNP, Single Nucleotide Polymorphism database; P, probably damaging; PCR, polymerase chain reaction; Po, polymorphism; Pos, possibly damaging; T, tolerated.

^a^The gene symbol is approved by the Human Genome Organisation Gene Nomenclature Committee.

^b^Predicted by Sorting Intolerant from Tolerant/Polymorphism Phenotyping version 2/MutationTaster.

^c^Lowercase letters in the primer sequences are 5'-end tags (forward and reverse primers) or non-homologous sequences (extending primers) added to increase the molecular weights.
